# Variations in Incidence and Prevalence of Parkinson's Disease in Taiwan: A Population-Based Nationwide Study

**DOI:** 10.1155/2016/8756359

**Published:** 2016-01-19

**Authors:** Chih-Ching Liu, Chung-Yi Li, Pei-Chen Lee, Yu Sun

**Affiliations:** ^1^Department of Public Health, College of Medicine, National Cheng Kung University, Tainan 70101, Taiwan; ^2^Department of Public Health, College of Public Health, China Medical University, Taichung 40402, Taiwan; ^3^Department of Health Care Management, National Taipei University of Nursing and Health Sciences, Taipei 11219, Taiwan; ^4^Department of Neurology, En Chu Kong Hospital, Sanxia District, New Taipei City 23702, Taiwan

## Abstract

Demographic, socioeconomic, and urbanization level variations in Parkinson's disease (PD) are rarely investigated, especially in Asia. This study describes an eight-year trend in PD incidence and prevalence in Taiwan as well as assessing the effects of sociodemographics and urbanization on the incidence and prevalence of PD. The data analyzed were acquired from the Taiwan National Health Insurance Research Database (NHIRD) entries between 2002 and 2009. The calendar year, sex, and age-specific rates were standardized, and the effects of the sociodemographics and urbanization on PD were assessed using Poisson regression analysis. PD incidence and prevalence showed a significantly increasing trend, with a greater magnitude noted for prevalence than for incidence (87.3% versus 9.2%). The PD incidence and prevalence increased with age and were slightly higher in men than in women. The people who were not under the labor force (i.e., dependents) or with lower monthly incomes were at significantly increased adjusted incidence rate ratio (1.50–1.56) and adjusted prevalence rate ratio (1.66–1.71) of PD. Moreover, significantly higher PD incidence and prevalence were noted in areas with lesser urbanization. This information emphasizes the need for preventive and clinical care strategies targeting the segment of Taiwanese population that exhibited a greater incidence and prevalence of PD.

## 1. Introduction

Parkinson's disease (PD) is the second leading neurodegenerative disorder with unknown etiology [1]. It influences about 1%-2% of the population aged over 65 years [2]. Many studies reported that the prevalence of PD had increased over the past decades, partially attributed to the increasing older population worldwide [3–8]. By contrast, most studies showed no substantial changes in the incidence of PD over time [4, 5, 7, 9, 10]. Moreover, race/ethnic variations in PD incidence and prevalence have been reported [11]. Previous studies estimated that the crude incidence and prevalence rates of PD in European countries range from 5 per 10^5^ person-years to 346 per 10^5^ person-years and from 65.6 per 10^5^ population to 12,500 per 10^5^ population, respectively [11, 12]. Unlike the figures reported in Western nations, the incidence and prevalence of PD are relatively low in Asia [11, 13]. However, these Asian data were obtained from a small number of studies, and most of these Asian studies were based on regional community surveys or information from selected hospitals [13–16], which are subject to potential nonrepresentativeness.

Genetic variants are considered to be the most likely etiological factor for PD incidence in patients <40 years [17], and certain occupational and environmental factors add additional risk to the incidence of PD [18–20]. Sufficient evidence shows that age is the strongest risk factor for PD [19], where the PD incidence starts to increase sharply after 60 years of age [1]. Despite this trend, several studies found that the incidence of PD increases up to a peak in 70–79 years and then declines in very old patients [12, 13, 21]. Such phenomenon may be attributed to the difficulty in distinguishing between normal and PD patients in the most advanced ages whose neurodegeneration may be regarded as “normal” aging-related signs [13].

Previous studies comparing the incidence and prevalence rates across urban/rural areas reported inconsistent findings [16, 20, 22–26]. Several studies showed that people with agricultural occupations, such as farmers in rural areas, have higher incidence rates of PD, which could be associated with their increased exposure to herbicides/pesticides relative to the general population [20]. Other studies reported the higher incidence rates of PD in urban than in rural areas, suggesting the possible links of PD with higher levels of pollutants/oxidative stress in cities [22] or to working on a construction site in urban areas [27], where exposure to certain chemicals is more frequent than in other areas [27, 28]. Similarly, a relationship was found between personal socioeconomic status (SES) and the PD incidence [4, 22, 29]. A Canadian study showed that SES is inversely associated with PD [4], which may reveal that people with lower SES are more likely to be employed by occupations involving exposure to certain environmental contaminants [30, 31]. Several studies from the UK [22], Sweden [30], and Italy [32] noted that people with higher SES experience an increased risk of PD. This result may be explained by lower physical activity [32] and higher PD coding practice in practitioners in areas with lesser social deprivation [22].

Taiwan is an Asian country with a rapid increase in the elderly population. The community-based studies in various areas of Taiwan [14–16] revealed the age-adjusted PD incidence and prevalence rates of 28.7 per 10^5^ person-years [15] and 112–633 per 10^5^ population, respectively, for people aged 40 years and over. A recent population-based study in Taiwan reported that the age-standardized incidence of PD decreased from 35.3 per 10^5^ person-years in 2005 to 28.8 per 10^5^ person-years in 2011, whereas the age-standardized prevalence of PD increased from 84.8 per 10^5^ to 147.7 per 10^5^ over the same period [8]. However, these previous studies covered the entire population including children and young adults, who are at very low risk of developing PD. Furthermore, the only population-based study utilized the 2005 population data of Taiwan as the standard population employed in age standardization, which did not allow direct comparison with figures from other nations. We therefore conducted this population-based study, using the World Health Organization (WHO) standard population, in an attempt to estimate the incidence and prevalence of PD in people aged 40 years and beyond from 2002 to 2009. This study investigated whether demographic, SES, and urbanization level variations exist in the PD incidence and prevalence in Taiwan.

## 2. Research Design and Methods

### 2.1. Data Source

We obtained our data from the Taiwan's National Health Insurance (NHI) database, which has been routinely collected by the National Health Research Institutes and is supervised by the National Health Insurance Administration (NHIA), Ministry of Health and Welfare. A universal NHI program has been implemented in Taiwan since March 1995. By the end of 2008, more than 99% of the total Taiwanese population (about 23 million people) had enrolled in the NHI program [33]. By the end of 2009, the NHIA had made a contract with 92.5% of hospitals and clinics throughout the nation [34]. The NHIA performs quarterly expert reviews on a random sample of every 50–100 ambulatory and inpatient claims in each hospital and clinic to ensure the accuracy of the claims data [35]. The NHI data sets were among the largest and most comprehensive data sets in the world and have been used in many published epidemiologic studies on PD [36–39].

In Taiwan, PD is usually diagnosed by neurologists, and the PD diagnosis included in the NHI claims is considered valid [40]. We used the NHI medical claims data of ambulatory care claims (1999–2009), details of ambulatory care orders (1999–2009), all inpatient claims (1999–2009), details of inpatient orders (1999–2009), and the updated registry for beneficiaries (2002–2009), with the ethical approval of the National Health Research Institutes. All data sets can be interlinked through each individual's encrypted personal identification number. The Institutional Review Board of the Taipei City Hospital approved this study (Number TCHIRB-1020702-E).

### 2.2. Study Subjects

In this study, the patients were regarded as having PD if they had at least three medical claims (either ambulatory or inpatient care) with a diagnostic code of PD (International Classification of Disease, 9th Revision, Clinical Modification (ICD-9-CM) code 332.0) and they had received anti-Parkinsonism medications, including L-dopa or dopamine agonist prescriptions, three or more times after the first-time diagnosis between 2002 and 2009. Moreover, the first and last outpatient or inpatient visits and anti-Parkinsonism medication records should be separated by at least 90 days to avoid accidental inclusion of miscoded patients. To include only the first-time diagnosed PD cases (i.e., incident cases), we excluded those who had a prior diagnosis of PD in 1999 to 2001. The first date of initial diagnosis for PD in the period of 2002 to 2009 was set as the index date.

We further applied the following exclusion criteria to ensure the validity of the PD diagnosis: (1) age younger than 40 years on the index date, because PD with onset age younger than 40 is rare, and miscoding encephalitis or some other genetic disorder such as Wilson's disease mimicking young onset PD is probably likely; (2) being given a diagnostic code of secondary Parkinsonism (ICD-9-CM code: 332.1) during the study period; (3) receipt of any neuroleptic medication within 180 days prior to the index date, and (4) being with three or more medical claims (either ambulatory or inpatient care) having the diagnostic codes of dementia (ICD-9-CM code: 290, 331) prior to the index date.

Patients who obtained a PD diagnosis prior to 2002 and those who developed PD and were alive in the subsequent years after the PD incidence were considered as prevalent cases. With the above inclusion and exclusion criteria, this study obtained 26,996 patients with first-time PD diagnoses and 181,277 prevalent PD accumulated in 2002–2009.

### 2.3. Demographics, SES, and Urbanization Level

Information on demographic- and SES-related factors, including age, sex, and salary-based insurance premium, as well as on the level of urbanization, was obtained from beneficiary records (2002–2009) of the National Health Insurance Research Database (NHIRD). The patients were categorized on the basis of age in years at first PD diagnosis (for incident cases) or at mid-year (for prevalent cases) into five groups as follows: 40–49, 50–59, 60–69, 70–79, and ≥80. The patients were also grouped on the basis of salary-based insurance premium into three levels: dependents, less than median in each year, and greater than or equal to median. We categorized the PD patients on the basis of living area into three levels of urbanization in accordance with the classification scheme proposed by Liu et al. [41], who considered the following indicators in determining levels of urbanization: population density, proportion of residents with college or higher education, percentage of elderly (>65 years) people, proportion of agriculture workforce, and number of physicians per 10^5^ population.

### 2.4. Validation

We validated the diagnosis of PD in NHI claims by reviewing the charts of 290 patients who were randomly selected from all PD patients treated in En Chu Kong Hospital, a tertiary referral center in northern Taiwan, between January and October 2012. Among the randomly selected 290 patients coded with PD, 245 were confirmed by chart review as suitable, whereas 6 were confirmed not suitable, for all the eligibility requirements of PD. The sensitivity, specificity, positive predictive value, and negative predictive value of our method for identifying PD cases were 97.6%, 92.3%, 98.8%, and 85.7%, respectively. However, the information obtained from the above validation may not necessarily be generalized to all medical institutions in Taiwan.

### 2.5. Statistical Analysis

The biannual crude incidence and prevalence rates were estimated by dividing the number of incidence and prevalent cases of PD, respectively, by the total number of people in the NHI program in every two years from 2002 to 2009. We further calculated, using the WHO 2000 standard population, age-sex-standardized incidence and prevalence rates of PD over time [42]. The Poisson regression was employed to test whether a linear secular trend in PD incidence/prevalence exists over the study period. The age-standardized incidence and prevalence rates of PD over time were also stratified according to sex, and the age- and sex-specific incidence/prevalence rates were calculated from the weighted average of the annual incidence/prevalence rates of age and sex stratifications.

To account for the independent effects of age, sex, and calendar year as well as the potential effects of urbanization and SES indicated by insurance premium on PD incidence/prevalence, we conducted a multivariate Poisson regression analysis. The generalized estimation equation (GEE) method was used to account for the possible clustering of data collected from the PD cases living with the same level of urbanization and counted repeatedly in estimating the prevalence rate, which may yield robust standard error associated with the risk estimates [43]. The statistical analysis was performed using SAS version 9.4 (SAS Institute, Cary, NC, USA). *p* < 0.05 was considered statistically significant.

## 3. Results

**Figure 1:**
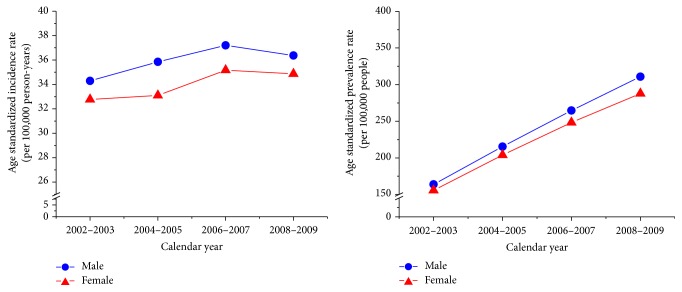
Age-standardized prevalence and incidence rates of Parkinson's disease in Taiwan, 2002–2009.

**Figure 2:**
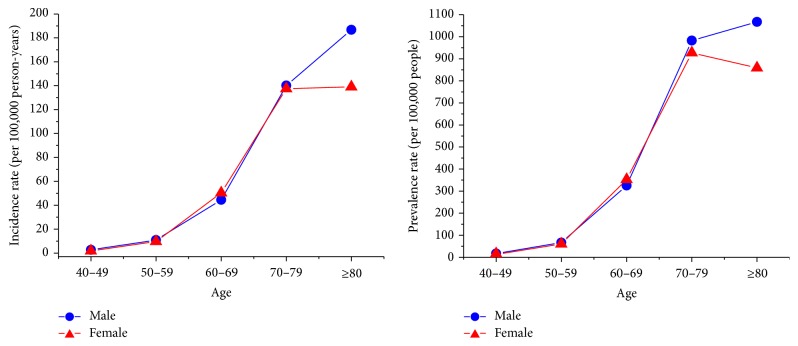
Age- and sex-specific incidence and prevalence rates of Parkinson's disease in Taiwan, 2002–2009.

The mean ± standard deviation of the age at PD diagnosis was 72.9 ± 9.7 years for men and 72.0 ± 9.3 years for women. The age-sex-standardized incidence of PD moderately increased from 33.5 to 36.6 per 10^5^ person-years between 2002 and 2009, whereas the age-sex-standardized prevalence rose substantially from 159.8 to 299.3 per 10^5^ population, representing a 1.88-fold increase within the same period (Table 1). Similar secular trends were noted for both men and women (Figure 1). The age-specific incidence and prevalence rates for both men and women started to markedly increase at the age of 60 years. However, unlike men whose incidence and prevalence rates continually increased at extremely advanced ages, the women appeared to peak their incidence and prevalence rates at ages 70–79 and then the prevalence rates decline at ages 80 and older (Figure 2).

Compared with that of 2002-2003, the incidence of PD steadily increased in the subsequent years, with an adjusted incidence rate ratio (AIRR) ranging from 1.09 to 1.22. The adjusted prevalence rate ratio (APRR) also showed an increasing trend, with greater figures from 1.33 to 1.94. Age appeared to affect PD incidence and prevalence to a greater extent than sex. Compared with those aged 40–49 years, older people experienced notably higher risks of PD incidence and prevalence. A positive linear trend also existed in both AIRR and APRR across age groups. No significant difference in PD incidence was noted between genders. Although a significantly increased APRR was found for men, the magnitude of increase was small (APRR = 1.08, 95% confidence interval (CI) = 1.02–1.14).

In addition to demographic characteristics, Table 2 also shows the influences of salary-based insurance premium and level of urbanization on the incidence and prevalence of PD. Compared with those with higher insurance premium (≥median), the patients with lower-than-median insurance premium (AIRR = 1.56, 95% CI = 1.36–1.79) and those who were dependents (AIRR = 1.50, 95% CI = 1.39–1.63) had significantly higher incidence rates of PD. They also had a significantly increased APRR of PD. Additionally, significant urbanization level variations existed in PD incidence and prevalence, in which people from rural areas showed the greatest AIRR (1.11, 95% CI = 1.05–1.18) and APRR (1.21, 95% CI = 1.13–1.29) compared with urban residents.

## 4. Discussion

### 4.1. Main Findings

This study demonstrated the significant demographic, SES, and urbanization level variations in the incidence and prevalence of PD in people aged ≥40 years in Taiwan. The results showed that older age, lower insurance premium, and lower level of urbanization were significant predictors for the higher incidence and prevalence of PD. Male gender was also a significant predictor for PD prevalence but not for PD incidence.

The standardized PD incidence rate noted in our studies was generally higher than the figures reported in the earlier studies of Taiwan, Japan, China, Singapore, and Spain but was comparable with the data from Italy [13]. Similar comparative results were observed for the PD prevalence rate. Nonetheless, obtaining comparisons of the findings between the present and previous studies is difficult, if not impossible, mainly because of the dissimilarities in data sources (medical claims versus door-to-door survey), PD diagnostic and age criteria, and study duration.

### 4.2. Trends of the Incidence and Prevalence of PD

Our study showed a moderate but significant increase in PD incidence in Taiwan, which is inconsistent with the reports from previous studies that presented a stable [4, 5, 7, 9, 10] or decreasing trend [8, 22]. Studies conducted in Japan (1992 and 2004) [5], Argentina (2003 and 2008) [9], Indian Navajo Nation of the US (2002–2004 and 2009–2011) [10], and France (2005 and 2010) [7] all demonstrated relatively stable PD incidences. A decreasing trend in incidence of PD was observed in a UK study reporting an average reduction by 6% annually from 1999 to 2009 [22]. A recent Taiwanese study also showed a decreasing trend in PD incidence from 2004 to 2011 [8]. Discrepancy in a secular trend in PD incidence between previous studies and ours could be due to dissimilarities in age composition of study subjects or in the criteria of PD ascertainment [4, 5, 7–10, 22]. In particular, unlike our study that included only the population aged 40 years and older, the recent Taiwanese study mentioned above [8] included all-age population. In addition, the decreasing trend noted in the UK study may be attributed to the changes in PD diagnosis over time and possibly because of the greater recognition of atypical Parkinsonian disorders [22].

Previous studies suggested that aging, exposure to environmental toxins [19], mainly pesticides, metals, and solvents, and clinical factors, such as diabetes [36], could increase the risk of developing PD. An increase in PD incidence noted in this study could also be attributed to the dramatic increase in the prevalence of diabetes in Taiwan [44]. Additionally, industrialization over the past decades in Taiwan instigated the increase in the release of heavy metals, such as lead and manganese, and organic solvents from occupational settings [45], which could also contribute to the increase in PD incidence [45]. A slight increase in PD incidence and improved survival associated with the advancement of PD treatment accounted for an evident increase in PD prevalence.

### 4.3. Demographics, SES, and Urbanization Level Variations in PD Incidence and Prevalence

Inconsistent findings have been reported concerning the gender difference in PD incidence. The neuroprotective effects of estrogen in women, the higher chance of chemical exposure in men, and the recessive susceptibility genes on chromosome X have been hypothesized to possibly lead to a higher risk of PD in men [19, 23, 46]. Most studies conducted in Western countries showed that PD is more common in men than in women, but some Asian studies reported no such gender difference [13, 47]. Similarly, our study showed no significant gender difference in PD incidence. Although our study showed a significantly higher prevalence of PD in men, the magnitude of elevated prevalence was considered small.

Our study noted that lower SES was associated with an increased risk of developing PD, which is consistent with previous findings from Canada [4]. People who possess a number of risk factors for PD incidence, including jobs involving metal works, such as welding; exposure to metals, such as manganese and lead; or working as a farmer, usually exhibit lower SES [4]. Despite this trend, it cannot be clearly confirmed whether the observed association between lower SES and higher PD incidence/prevalence noted in this study is attributable to certain occupational hazards, simply because no occupational data are available from the NHI medical claims.

With regard to the urban-rural difference in PD incidence and prevalence, several studies suggested a significantly higher incidence of PD in subjects residing in rural areas [23], but others reported an increased incidence [22, 24, 25] and prevalence [16, 25] of PD in urban areas. Moreover, one study showed no such urban-rural difference [26]. The possible reasons for such discrepancy possibly include the differences in the definition of urbanization components and the classification of urbanization levels between studies [16, 22–26]. Additionally, previous studies included different ethnic populations [25] and were involved in various distributions of certain environmental and occupational risk factors for PD [16, 22–26], which could all contribute to the inconsistent results.

Interpretation of the SES and urbanization level variations in PD incidence and prevalence can be multifaceted. Exposure to certain occupational and environmental contaminants, such as pesticides, solvents, and metals, may increase the risk of PD [30, 31]. Individuals with lower SES and who are living in rural areas are more likely to have higher chances of exposures to the aforementioned contaminants. A case-control study conducted in Taiwan showed that residence in a rural environment and farming were associated with increased risk of PD incidence, suggesting that exposure to occupational herbicide/pesticide may have contributed to the observed association [23]. PD may also be associated with use of well waters in rural or farm areas [20, 28]. Previous occupational studies also found that exposure to metals, such as lead and manganese; solvents, such as fuels, paints, printing inks, degreasers, and cleaning products; and pesticides at workplaces was associated with a number of PD-related pathologic processes, including mitochondrial dysfunction, alterations in metal homeostasis, and aggregation of proteins, such as *α*-synuclein [18].

### 4.4. Methodological Concerns and Conclusion

This work is the first nationwide population-based study covering the entire population aged ≥40 years in Taiwan. The secular trend in PD incidence and prevalence and the effects of demographics, SES, and urbanization on the incidence and prevalence of PD in Taiwan were investigated. The study sample was considered highly representative. However, our study was limited by the following issues. First, we solely obtained our PD cases on physician-recorded diagnoses and prescription-reported medical claims, which may still be subject to potential disease misclassification. To avoid the accidental inclusion of miscoded patients, we involved only the PD patients who made three ambulatory or inpatient visits with PD diagnosis and prescriptions, with the first and last visits being more than 90 days apart, during the study period. We believe that this strategy would have greatly reduced the likelihood of disease misclassification. Moreover, the PD patients are usually treated by neurologists on a regular basis in Taiwan, leaving small room for the error on PD diagnosis [40]. Second, personal information including family history, lifestyle habits, and occupational data, which may contribute to the development of PD, are unavailable in the NHIRD. Lack of these data and information limits our explanation for present results.

We conclude that despite only a moderate increase in PD incidence from 2002 to 2009 in Taiwan, the prevalence of PD almost doubled over the study period. Given the significant socioeconomic and urbanization level variations in PD incidence and prevalence, further studies are warranted to investigate whether such findings can be attributed to specific occupational and environmental risk factors most relevant to the incidence and prevalence of PD in Taiwan.

## Figures and Tables

**Table 1:** Secular trends of the incidence and prevalence rates of Parkinson's disease in Taiwan, 2002–2009.

Variables	Incidence (per 100,000 person-years)^‡^					Prevalence rate (per 100,000 population)^‡^				
	Calendar year				Change^†^ (%)	Calendar year				Change^†^ (%)
	2002-2003	2004-2005	2006-2007	2008-2009		2002-2003	2004-2005	2006-2007	2008-2009	
Crude rate	34.3	35.2	36.9	36.6	6.9	164.1	214.5	260.6	306.8	87.0
Standardized rate^**‡**^	33.5	34.5	36.2	36.6	9.2	159.8	209.7	256.5	299.3	87.3

^†^Change (%): percentage of changes in the incidence and prevalence rates of Parkinson's disease between 2002 and 2009.

^‡^Adjusted for age and sex.

**Table 2:** Independent effects of calendar year, age, sex, insurance premium, and urbanization on the incidence and prevalence of Parkinson's disease, 2002–2009.

Variables	Incidence rate				Prevalence rate			
	Crude IRR	95% CI	Adjusted IRR^†^	95% CI	Crude PRR	95% CI	Adjusted PRR^†^	95% CI
Calendar year								
2002-2003	1.00		1.00		1.00		1.00	
2004-2005	1.03	0.99–1.06	1.09^*∗*^	1.07–1.11	1.31^*∗*^	1.29–1.33	1.33^*∗*^	1.29–1.37
2006-2007	1.08^*∗*^	1.04–1.11	1.19^*∗*^	1.16–1.22	1.59^*∗*^	1.57–1.61	1.64^*∗*^	1.57–1.70
2008-2009	1.07^*∗*^	1.03–1.11	1.22^*∗*^	1.18–1.27	1.87^*∗*^	1.84–1.90	1.94^*∗*^	1.88–2.00
	Trend test: *β* = 0.024, *p* < 0.0001		Trend test: *β* = 0.0672, *p* < 0.0001		Trend test: *β* = 0.2018, *p* < 0.0001		Trend test: *β* = 0.2130, *p* < 0.0001	
Age (years)								
40–49	1.00		1.00		1.00		1.00	
50–59	4.60^*∗*^	4.22–5.02	4.26^*∗*^	3.79–4.78	4.25^*∗*^	4.11–4.40	3.81^*∗*^	3.51–4.14
60–69	21.23^*∗*^	19.58–23.02	16.59^*∗*^	14.04–19.59	22.76^*∗*^	22.06–23.48	16.29^*∗*^	13.25–20.03
70–79	62.08^*∗*^	57.38–67.15	44.35^*∗*^	38.69–50.84	64.01^*∗*^	62.10–65.99	42.30^*∗*^	36.15–49.49
≥80	72.81^*∗*^	67.17–78.91	52.24^*∗*^	47.19–57.82	64.55^*∗*^	62.56–66.61	41.48^*∗*^	35.75–48.12
	Trend test: *β* = 0.9518, *p* < 0.0001		Trend test: *β* = 0.8998, *p* < 0.0001		Trend test: *β* = 0.9302, *p* < 0.0001		Trend test: *β* = 0.8631, *p* < 0.0001	
Sex								
Male	1.09^*∗*^	1.06–1.11	1.05	0.98–1.13	1.09^*∗*^	1.08–1.10	1.08^*∗*^	1.02–1.14
Female	1.00		1.00		1.00		1.00	
Insurance premium (NTD)								
Dependents	3.13^*∗*^	3.04–3.22	1.50^*∗*^	1.39–1.63	3.45^*∗*^	3.42–3.49	1.66^*∗*^	1.49–1.85
<Median (19,200)	2.46^*∗*^	2.38–2.54	1.56^*∗*^	1.36–1.79	2.67^*∗*^	2.64–2.71	1.71^*∗*^	1.51–1.94
≥Median	1.00		1.00		1.00		1.00	
Urbanization								
Urban	1.00		1.00		1.00		1.00	
Satellite	1.20^*∗*^	1.17–1.24	1.06^*∗*^	1.04–1.09	1.22^*∗*^	1.21–1.24	1.11^*∗*^	1.08–1.13
Rural	1.52^*∗*^	1.45–1.58	1.11^*∗*^	1.05–1.18	1.55^*∗*^	1.52–1.58	1.21^*∗*^	1.13–1.29

Note: inconsistency between the total population and population summed for individual variables was due to missing information.

^†^Estimated from the multivariate Poisson regression with GEE model, which simultaneously included the calendar year, age, sex, insurance premium, and urbanization.

^*∗*^ *p* < 0.05.

IRR: incidence rate ratio; PRR: prevalence rate ratio; CI: confidence interval.

NTD: New Taiwan Dollar; 1 USD ≈ 30 NTD.
